# Nutrition Care Process Model Approach to Surgical Prehabilitation in Oncology

**DOI:** 10.3389/fnut.2021.644706

**Published:** 2021-06-24

**Authors:** Chelsia Gillis, Leslee Hasil, Popi Kasvis, Neil Bibby, Sarah J. Davies, Carla M. Prado, Malcolm A. West, Clare Shaw

**Affiliations:** ^1^Department of Anesthesia, McGill University, Montreal, QC, Canada; ^2^Department of Nutrition Services, Alberta Health Services, Calgary, AB, Canada; ^3^Department of Nutrition, McGill University Health Center, Montreal, QC, Canada; ^4^Manchester Royal Infirmary, Dietetics Department, Manchester University National Health Service (NHS) Foundation Trust, Manchester, United Kingdom; ^5^Department of Dietetics/Speech and Language Therapy, University Hospital Southampton National Health Service (NHS) Foundation Trust, Southampton, United Kingdom; ^6^Department of Agricultural, Food and Nutritional Science, University of Alberta, Edmonton, AB, Canada; ^7^School of Cancer Sciences, Faculty of Medicine, University of Southampton, Southampton, United Kingdom; ^8^University Hospital Southampton National Health Service (NHS) Foundation Trust, Southampton, United Kingdom; ^9^National Institute for Health Research (NIHR) Biomedical Research Centre, University Hospital Southampton National Health Service (NHS) Foundation Trust, Southampton, United Kingdom; ^10^Anaesthesia, Perioperative and Critical Care Research Group, National Institute for Health Research (NIHR) Biomedical Research Centre, University Hospital Southampton National Health Service (NHS) Foundation Trust, University of Southampton, Southampton, United Kingdom; ^11^Department of Nutrition and Dietetics, The Royal Marsden National Health Service (NHS) Foundation Trust, London, United Kingdom

**Keywords:** surgical nutrition, oncological nutrition, pre-operative, pre-surgery, pre-habilitation, before surgery

## Abstract

The nutrition care process is a standardized and systematic method used by nutrition professionals to assess, diagnose, treat, and monitor patients. Using the nutrition care process model, we demonstrate how nutrition prehabilitation can be applied to the pre-surgical oncology patient.

## Nutrition Care Process

The nutrition care process model (NCPM) is a standardized and systematic approach that nutrition professionals, namely dietitians (referred to as Registered Dietitians, RDs, in most of Canada and the United Kingdom, and Registered Dietitian Nutritionists, RDN, in the United States), use to provide care (1). The NCPM has been adopted by international dietetic associations and is updated by an international working group every 5 years (2–5). The model follows nutrition screening and consists of four interrelated steps: (1) nutrition assessment, (2) nutrition diagnosis, (3) nutrition intervention and (4) nutrition monitoring and evaluation (1). The first two steps involve problem identification, while the final two steps involve problem solving. The structured framework was designed to enhance quality of care and nutritional status. Indeed, reported benefits of adopting the NCPM include enhanced productivity, improved resolution rate of nutrition-related problems, and improved physician acknowledgment of nutrition recommendations (6).

A recent scoping review of nutrition within prehabilitation oncology research identified that nutrition assessment was inconsistently applied across these studies, interventions did not often meet reference standards, and two-thirds of these studies did not monitor the nutrition intervention nor evaluate nutrition outcomes (7). Given that NCPM represents a global standard for provision of nutrition care, we advocate for its use in prehabilitation and have applied this model to the pre-operative surgical patient to illustrate how nutrition care can be effectively implemented and optimized.

### Nutrition Screening

Nutrition screening precedes the NCPM and is the first step in identifying subtle or overt malnutrition. Screening should be applied to all patients with cancer (8). Nutrition screening tools were designed to be administered quickly by non-nutrition professionals to identify patients *at risk of malnutrition*. Patients identified as being “at risk” would trigger a referral to a RD for a comprehensive nutrition assessment and diagnosis of malnutrition. Early screening at the first hospital appointment before surgery, or at minimum by the first surgical visit, using a validated tool, offers the opportunity to intervene with a targeted or specialized nutrition intervention (alone or in combination with other approaches, such as exercise and psychological support/behavior change) that could improve patient outcomes (9). Remedial nutrition therapy for ~7-14 days before surgery has been found to improve post-operative outcomes (8), including length of stay (10), and serious complications (11, 12). However, some observational evidence suggests that a longer period of nutritional repletion is required to improve parameters of physical functioning in malnourished patients (13, 14). An earlier screen affords greater possibility for nutrition care management and success. Patients who screen negative for malnutrition risk preoperatively should be re-screened if their condition changes or on admission to hospital.

Nutrition screening tools that are commonly used in oncology or surgery settings are listed in Table 1. Most of these tools have been validated using “gold standard” nutrition *assessment* tools, used to diagnose malnutrition, including the Subjective Global Assessment (SGA) (37, 38) and the Patient-Generated Subjective Global Assessment (PG-SGA) (39). Appreciation of these screening tools necessitates an understanding of malnutrition. Although there is no accepted definition for malnutrition, the condition can be described as an unbalanced nutritional state, resulting from inadequate nutrient intake and/or altered nutrient requirements related to disease and treatment, that alters body mass, body composition and function (40, 41). Recently, the Global Leadership Initiative on Malnutrition (GLIM) (42) convened to offer expert-consensus on the core criteria to diagnose malnutrition in a clinical setting. This group described the diagnosis of malnutrition as having an etiology and a phenotype. The etiology includes reduced food intake/food assimilation, malabsorption, disease burden/inflammation, and the phenotype is expressed with weight loss, reduced muscle mass, and low body mass index. A diagnosis of malnutrition is based upon the presence of at least one phenotypic criterion and one etiologic criterion.

**Table 1:** A list of nutrition risk screening tools and their psychometric properties for use in oncology and surgical settings.

**Tool**	**Phenotype**	**Etiology**	**Psychometric properties and intended population**
Mini nutritional assessment—short-form (MNA-SF)	Unintentional weight loss Low BMI Low muscle mass	Reduced food intake Disease burden	As far as we are aware, this tool has not been validated against SGA or PG-SGA in surgical or oncological populations. However, this tool has been validated against the full MNA, which is a valid nutritional *assessment* tool used to diagnose malnutrition in older adults, specifically (15).
Malnutrition screening tool (MST)	Unintentional weight loss	Reduced food intake	Mixed cancer types, oncology inpatients, *n* = 126 (16): Sensitivity: 66% Specificity: 83% Positive predictive value: 91% Negative predictive value: 49% (as compared with the PG-SGA)
			Mixed cancer types, radiation, *n* = 106 (17); chemotherapy, *n* = 50 (18) and *n* = 246 (19); chemotherapy or supportive cancer care, *n* = 201 (20); outpatients, *n* = 300 (21): Sensitivity: 70.6-100% Specificity: 69.5-92% Positive predictive value: 40-59% Negative predictive value: 99-100% [as compared with the PG-SGA (18, 19, 21), SGA (17, 20)]
			Cancer and non-cancer, surgical inpatients, preoperative evaluation, *n* = 100 (22): Sensitivity: 54% Specificity: 25% Kappa coefficient: 0.90 (as compared with the SGA)
Malnutrition universal screening tool (MUST)	Unintentional weight loss Low BMI	Reduced food intake Disease burden	Mixed cancer types, radiation outpatients, *n* = 450 (23); chemotherapy outpatients, *n* = 100 (24): Sensitivity: 80-86.7% Specificity: 89-94.5% Positive predictive value: 87-92.9% Negative predictive value: 100-89.7% Kappa coefficient: 0.79-0.86(as compared with the PG-SGA)
			Colorectal cancer, surgical inpatients, preoperative assessment, *n* = 45 (25): Sensitivity: 96% Specificity 75% Positive predictive value: 82.8% Negative predictive value: 93.8% Kappa coefficient: 0.7(as compared with the SGA)
			Cancer and non-cancer, surgical inpatients, preoperative assessment, *n* = 300 (26), assessment performed within 36 h of admission, *n* = 120 (27): Sensitivity: 67.8-85% Specificity: 93-94.4% Positive predictive value: 76-89% Negative predictive value: 91.9-99%(as compared with the SGA)
			Cardiac, surgical inpatients, preoperative assessment, *n* = 894 (28): Sensitivity: 97.9% Specificity: 87.1% Positive predictive value: 29.7% Negative predictive value: 99.9%(as compared with the SGA)
Nutritional risk screening-2002 (NRS-2002)	Unintentionalweight loss Low BMI	Reduced food intake Disease burden	Head and neck/CNS cancer, oncology outpatients, *n* = 124 (29): Sensitivity: 67.5% Specificity: 92.9% Positive predictive value: 97.7% Negative predictive value: 68.4% Kappa coefficient: 0.71(as compared with the SGA)
			Gastric cancer, surgical inpatients, assessment performed within 24 h of admission, *n* = 80 (30): Sensitivity: 80% Specificity: 96% Kappa coefficient: 0.69(as compared with the SGA)
			Cancer and non-cancer, surgical inpatients, preoperative assessment, *n* = 300 (26), assessment performed within 36 h of admission, *n* = 120 (27): Sensitivity: 60.7-80% Specificity: 89-96.3% Positive predictive value: 80.9-87% Negative predictive value: 90.4-100%(as compared with the SGA)
Short nutrition assessment questionnaire (SNAQ)	Unintentional weight loss	Reduced food intake	Cardiac, surgical inpatients, preoperative assessment, *n* = 894 (28): Sensitivity: 91.5% Specificity: 87.5% Positive predictive value: 28.9% Negative predictive value: 99.5% (as compared with the SGA)
Canadian nutrition screening tool (CNST)	Unintentional weight loss	Reduced food intake	Inpatients, (on admission) 22% of sample surgical, *n* = 123 (31): Sensitivity: 72.9% Specificity: 85.9% Positive predictive value: 82.7% Negative predictive value: 77.5%(as compared with the SGA)
Royal Marsden Nutrition Screening Tool (RMNST)	Unintentional weight loss Underweight appearance	Reduced food intake Reduced food assimilation	Mixed cancer types, oncology inpatients, *n* = 126 (16): Sensitivity: 93% Specificity: 53% Positive predictive value: 83% Negative predictive value: 76%(as compared with the PG-SGA)
Abridged patient-generated subjective global assessment (aPG-SGA)	Unintentional weight loss	Reduced food intake Reduced food assimilation	Mixed cancer types, oncology outpatients, *n* = 246 (19), *n* = 300 (32), *n* = 90 (33): Sensitivity: 80.4-96.9% Specificity: 72.3-86.2% Positive predictive value: 45% (19) Negative predictive value: 98% (19) Kappa coefficient: 0.49 (19)[as compared with PG-SGA (19, 32) and the SGA (33)]
NUTRISCORE	Unintentional weight loss	Reduced food intake Reduced food assimilation	Mixed cancer types, oncology outpatients, *n* = 394 (34): Sensitivity: 97.3% Specificity: 95.9% Positive predictive value: 84.8% Negative predictive value: 99% Area under the curve: 0.95(as compared with the PG-SGA)
Bach Mai Boston Tool (BBT)	Unintentional weight loss Low BMI	Reduced food intake	Mixed cancer types, oncology outpatients, *n* = 270 (35): Sensitivity: 67.1% Specificity: 94.4% Positive predictive value: 93.3% Negative predictive value: 70.9% Area under the curve: 0.81 Kappa coefficient: 0.6(as compared with the PG-SGA)
Malnutrition screening tool for cancer (MSTC)	Unintentional weight loss Low BMI	Reduced food intake	Mixed cancer types, oncology inpatients, *n* = 1,057 (800 for development, 257 for validation) (36): Sensitivity: 94% Specificity: 84.2% Positive predictive value: 67.8% Negative predictive value: 97.6% Area under the curve=0.95 Kappa coefficient: 0.7(as compared with the PG-SGA)
Perioperative nutrition screen (PONS)	Unintentional weight loss Low BMI	Reduced food intake Disease burden	As far as we are aware, this tool has not been validated against SGA or PG-SGA in surgical or oncological populations

*BMI, body mass index; CNS, central nervous system; PG-SGA, Patient Generated Subjective Global Assessment; SGA, Subjective Global Assessment*.

Table 1 provides a list of nutrition risk screening tools, applies the GLIM criteria to these tools, and presents the psychometric properties of these tools to help the reader select the most appropriate tool for their patient population. Choice of an appropriate nutrition screening tool will depend on local factors including whether validation studies have been completed in the population of interest, sensitivity and specificity to detect malnutrition, prevalence of malnutrition, available resources, ease of completion and capacity for collecting data by healthcare professionals or patients themselves. Ideally a tool should be both highly sensitive and specific; however, a perfect screening tool does not exist. A tool with 75% sensitivity would identify 75% of malnourished patients correctly but 25% of malnourished patients would remain undetected (43). A tool with 75% specificity would correctly identify those *without* malnutrition 75% of the time, but 25% of the time a patient without malnutrition would be falsely labeled as being “at malnutrition risk” and thus referred to the RD for assessment unnecessarily (44). Given that a misdiagnosis of being at risk of malnutrition (i.e., false positive) is relatively benign if resources for a follow-up assessment by an RD are available, use of a highly sensitive tool is desirable. An institution with limited RD resources for follow-up assessment post-screening, however, might consider a tool that is highly specific to reduce the number of non-malnourished patients being referred to the RD for assessment (but in selecting this tool would accept that a portion of malnourished patients will remain undetected). For an excellent review of considerations for selecting screening tools we refer the reader to Elia and Stratton (45).

### Nutrition Assessment

Nutrition screening tools do not perfectly identify patients with malnutrition. A highly sensitive tool would correctly identify malnourished patients while a highly specific tool would correctly identify non-malnourished patients (44). Thus, patients who are identified as being at risk for malnutrition must receive a nutrition assessment. Nutrition assessments are conducted by a RD for the purpose of diagnosing malnutrition and other nutrition-related problems. Nutrition assessment is a “systematic approach to collect, classify, and synthesize important and relevant data” (1). RDs use validated malnutrition assessment tools, including the SGA and PG-SGA, to diagnose malnutrition. RDs also perform comprehensive nutrition assessments that involve an evaluation of food and nutrition-related history, anthropometric measurements, biochemical data, health and disease status, psychological and behavioral issues, social and environmental influences, and a nutrition-focused physical exam/functional assessment.

An assessment of food and nutrition-related history includes an evaluation of food records or dietary food recalls to estimate usual nutrient intakes and the adequacy of these intakes. The National Cancer Institute offers an excellent resource on choosing an appropriate tool for estimating usual nutrient intakes (https://dietassessmentprimer.cancer.gov/approach/). Considerations for selection of a dietary tool include whether the goal is simply to describe dietary patterns, assess dietary intake, examine an association, or to evaluate the effect of an intervention. When assessing the effect of an intervention, multiple 24-h recalls are often cited as the best estimate of usual intakes (46). Although new technologies, including mobile apps, may enhance the accuracy of food records (47). If the goal of the intervention is to change behavior, food records could be an appropriate tool to support and track behavior change (48).

An assessment of nutrition-related history also includes an evaluation of nutrition-impact symptoms, including loss of appetite and diarrhea, that impede adequate oral intake. A prospective longitudinal survey of the nutrition-impact symptoms experienced by patients undergoing systemic anti-cancer treatment (SACT) identified that three-quarters experienced at least 1 symptom that affected food intake, including dry mouth, nausea and constipation, within 1 and 6 months of starting chemotherapy and nearly half of these patients continued to experience symptoms 12 months later (49).

Biochemical assessments for nutritional status are largely non-specific and, as a result, nutrition diagnoses are rarely based on biochemical data alone, but rather should be used as a complement to a thorough examination (50). Hypoalbuminemia (low serum albumin concentration), for instance, is not necessarily indicative of malnutrition (i.e., a reduced synthesis of albumin due to reduced substrate availability) because this plasma protein is a negative acute phase reactant that is affected by several conditions including cancer. Albumin also has a long half-life, and thus does not reflect acute changes in nutritional status. However, albumin is predictive of morbidity and mortality (50). While prealbumin (the precursor to albumin) is also a negative acute phase reactant, its pool is smaller and its half-life is shorter, which might make it a more reliable indicator of nutritional status in patients without inflammation (e.g., elevated c-reactive protein) (50). However, few studies have evaluated its relevance in predicting patient prognosis. As such, prealbumin has not been recommended for the diagnosis of malnutrition (51). C-reactive protein is a commonly used inflammatory marker with several prospective studies suggesting it predicts mortality in cancer (52).

An evaluation of glycated hemoglobin (HbA1c) might be beneficial in patients with and without diabetes. A systematic review of non-diabetic surgical patients identified that 34% of this heterogenous sample had sub-optimal preoperative glycemic control (53) and several observational studies in pre-surgical patients without cancer have suggested that there is a link between preoperative glycemia and postoperative outcomes (53–55). Fructosamine (another index of glucose homeostasis) has a shorter half-life than HbA1c, and thus might be useful for the assessment of acute changes in the short period before surgery (56). Other biochemical assessments to consider include serum levels of micronutrients, such as 25-hydroxyvitamin D (57). Finally, many patients present to surgery with anemia, which is associated with higher rates of morbidity and mortality (58). Several recent reviews have suggested that correction of iron deficiency anemia with iron therapy should take place in the pre-operative/preadmission clinic as a standard component of medical optimization (58, 59). For this reason, integration of prehabilitation programs within pre-operative clinics is recommended (59).

An anthropometric assessment of weight (including weight change), height, and waist circumference are vital components of the comprehensive nutrition assessment. Additionally, body composition assessment has emerged as a crucial component in the evaluation of patients' nutritional status (60). For an excellent review of the methodologies and techniques available for body composition assessment please see Prado and Heymsfield (61). Bioelectrical impedance, when conducted using standardized methods, is often cited as a reasonable option for estimating body composition in a clinical setting, especially in the assessment of change over time (62).

Indirect measures of nutrition status include assessment of strength and function. Malnutrition incites adaptive mechanisms that reduce basal metabolic rate and diminish physical performance in an attempt to conserve nutrient reserves (63). As a result, reduced strength and function are associated with malnutrition status. For instance, using a standard protocol to measure handgrip strength [grip measured three times with a 15 s break between trials (64)], a malnourished patient might exhibit low age- and sex-specific strength or poor recovery between measurements (i.e., a drop in strength with each consecutive measurement) (64). Common methods for testing physical function include the 6-min walk test, gait speed, Short Physical Performance Battery, timed up and go, and 30-s sit-to-stand (62, 65).

### Nutrition Diagnosis

Collected data from the nutritional assessment are compared against accepted standards, expert recommendations, and/or patient-defined goals to ascertain nutritional status (1). The aforementioned information, together with the patient's medical and social history, is used to diagnose nutrition-related problems that can be solved by the RD.

Table 2 lists surgery and oncology-specific accepted standards and/or recommendations from several nutrition associations, including the European Society for Clinical Nutrition and Metabolism (ESPEN). We have limited this list to general recommendations and guidelines in surgery and oncology, but the reader should be aware that many disease-specific standards also exist (75). Unfortunately, accepted nutrition guidelines are not often used in prehabilitation (7). A scoping review of 37 prehabilitation studies with a nutrition treatment component in oncology identified that only half of these studies (*n* = 21) specified a goal for their nutrition intervention; of these, 67% (*n* = 14) referenced the stated goals and only 43% (*n* = 9) used a reference standard or accepted guideline, including ESPEN guidelines (7). The potential to improve patient outcomes is limited unless clinical guidelines are followed (76).

**Table 2:** Clinical nutrition guidelines for surgery and/or oncology patients.

**Organization**	**Energy requirements**	**Protein requirements**	**Screening/assessment tool**
European society of enteral and parenteral nutrition (ESPEN)			
Oncology (66)	25-30 kcal/kg/day	>1-1.5 g/kg/day	Screening: NRS-2002, MUST, MST Assessment: SGA, PG-SGA, MNA
Surgery (8)	25-30 kcal/kg/day	1.5 g/kg/day	Screening: NRS-2002 Assessment: SGA
Clinical oncology society of Australia (COSA) (67)	25-30 kcal/kg/day	1-1.5 g/kg/day	MST, MUST, MSTC, abPG-SGA
French Speaking Society of Clinical Nutrition and Metabolism (SFNEP) (68)	30-35 kcal/kg/day	1.2-1.5 g/kg/day	All patients: PG-SGA, SGA Geriatric patients: MNA
Polish societies of: surgical oncology, oncology, clinical oncology and parenteral, enteral nutrition and metabolism (69)	25-35 kcal/kg/day 35-45 kcal/kg/day (severe cachexia)	0.8-1.5 g/kg/day 2-3 g/kg/day (severe cachexia)	SGA, NRS-2002, MUST Geriatric patients: MNA
Spanish society of medical oncology (SEOM) (70)	25-30 kcal/kg/day	1.2-1.5 g/kg/day	Outpatients: MUST Inpatients: NRS-2002 Geriatric patients: MNA-SF In/outpatients: assessment MST PG-SGA
Oncology evidenced-based nutrition practice guidelines for adults (71)	No recommendation	No recommendation	Inpatient: MST, MSTC, MUST Outpatient: MST
Nutritional support and parenteral nutrition in cancer patients: an expert consensus report (72)	25-30 kcal/kg/day	1-2 g/kg/day 1-1.2 g/kg/day for patients with acute/chronic renal failure	MST, PG-SGA
American Society for Enhanced Recovery and Perioperative Quality Initiative (73)	25-30 kcal/kg/day	>1.2-2.0 g/kg/day	PONS
Enhanced recovery after surgery society (ERAS) and the European society of surgical oncology (ESSO)-Gastrointestinal cancers (74)	25-30 kcal/kg/day	1.5 g/kg/day ideal body weight	PG-SGA

*abPG-SGA, abridged scored Patient Generated Subjective Global Assessment; MNA, Mini Nutritional Assessment; MSTC, Malnutrition screening tool for cancer; MUST, Malnutrition universal screening tool; NRS-2002, Nutritional risk screening-2002; PG-SGA, Patient Generated Subjective Global Assessment; PONS, Perioperative nutrition screen; SGA, Subjective Global Assessment*.

Based on the comprehensive nutritional assessment, the RD identifies a nutrition-related problem that can be treated (1). This diagnosis is expressed using standardized language by labeling the identified problem, citing the etiology of the problem, and providing evidence of the problem (i.e., signs and symptoms). Malnutrition is a common nutrition diagnosis pre-surgery. The prevalence of malnutrition in oncological patients is reported to range from 10 to 85% (77), depending on the definition of malnutrition, assessment tool, tumor-type, cancer stage, and adjuvant/neoadjuvant treatments (78). An example diagnostic statement pre-surgery is as follows: severe chronic malnutrition (problem) related to nutrition-impact symptoms, including constipation, early satiety and fatigue (etiology) as evidenced by meeting 65% of estimated protein requirements, 10% weight loss in past 6 months, and low handgrip strength. Other common nutrition diagnoses pre-surgery include inadequate oral intake, inadequate protein energy intake, impaired nutrient utilization, altered gastrointestinal function, unintended weight loss, underweight, and food and nutrition related knowledge deficit. Although not part of the NCPM standard terminology, a diagnosis of sarcopenia, which can occur independently of malnutrition (41, 79), is also an important nutrition-related diagnostic consideration given the catabolic impact of surgery. Sarcopenia in cancer can be primary (aging related), secondary (disease related) or both. These differences are important as primary sarcopenia is defined as depleted muscle mass and strength, while secondary sarcopenia is defined as only a measure of depleted muscle mass [the latter is an approach used in the vast majority of oncology-related publications on the topic (79, 80)].

### Nutrition Intervention

The NCPM defines a nutrition intervention as “a purposefully planned action(s) designed with the intent of changing a nutrition-related behavior, risk factor, environmental condition, or aspect of health status” (1). The nutrition intervention is designed to improve or resolve the nutrition diagnosis/problem. If it is not possible to resolve the diagnosis or its etiology, the nutrition plan is aimed at relieving signs and symptoms. Importantly, for patients who have been assessed by an RD and diagnosed with a nutrition problem, there is no “one-size-fits-all” approach to resolve the problem. Instead, the comprehensive nutrition assessment and diagnosis are used to guide a personalized intervention. As an example, a diagnosis of “inadequate oral intake related to nausea” would require an intervention to improve the diagnosis of inadequate oral intake based on treating its etiology of nausea, and with consideration of the patient's own goals, food preferences, capacity to prepare meals, food and nutrition knowledge, health literacy, and motivation to change.

**Figure 1:**
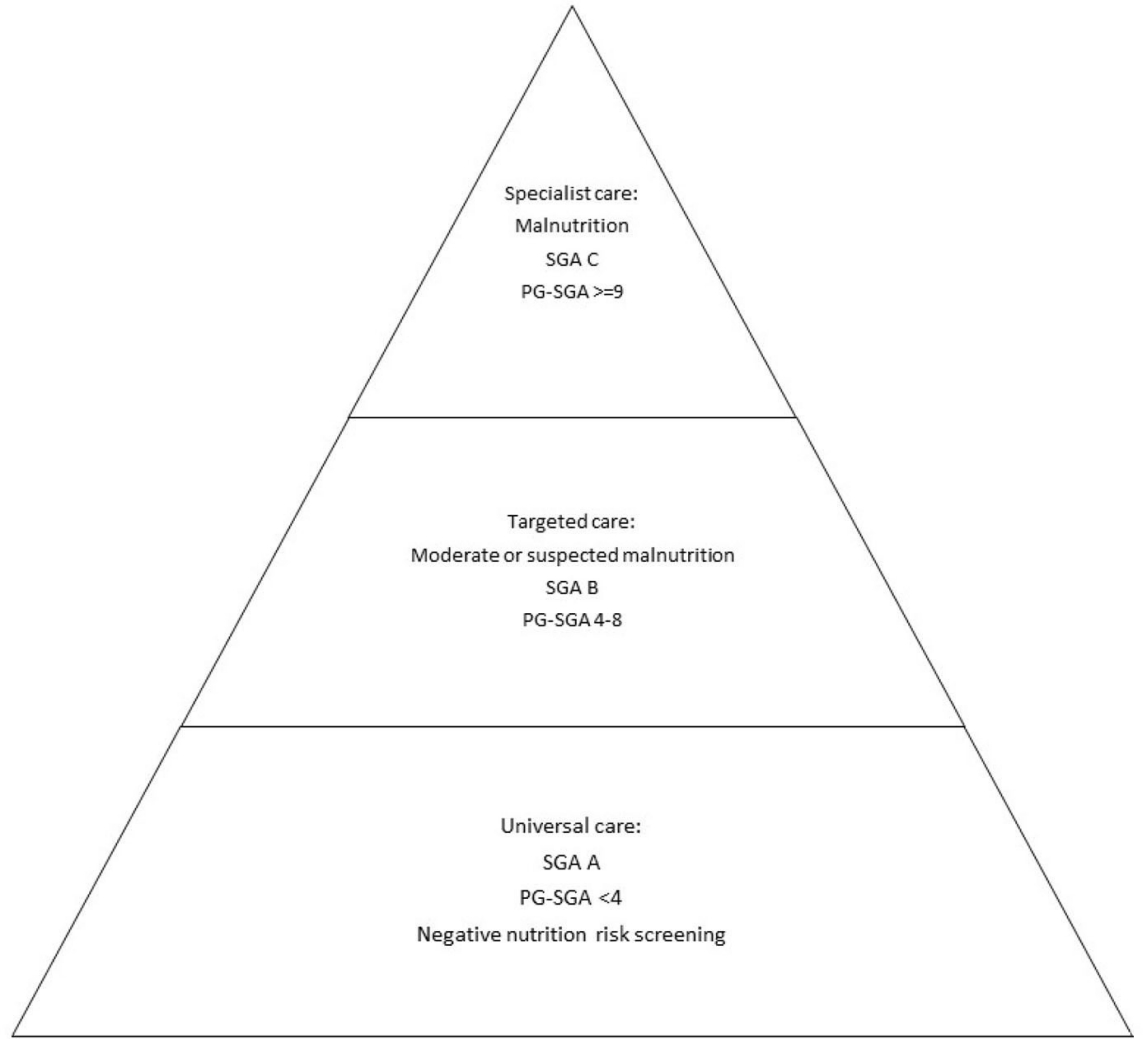
Risk stratified care for nutrition prehabilitation.

The first principles and guidance for the conduct of multimodal prehabilitation in cancer were released in 2019 by Macmillan Cancer Support, the Royal College of Anaesthetists and the National Institute of Health Research Cancer and Nutrition Collaboration; this guideline proposed that prehabilitative care should be delivered on a risk-stratified basis to use resources wisely (81). Using this approach, each patient's level of care is based on whether their assessment revealed that a minimal (targeted) intervention or a more intensive (specialist) intervention is needed. Using our experience with prehabilitation (14), we have modified the risk stratified diagram to suit nutrition prehabilitation (Figure 1). A patient who has been screened (using tools listed in Table 1) and is not at risk of malnutrition or has been assessed by a RD and is not malnourished (i.e., SGA A or PG-SGA < 4), would not require further assessment, diagnosis, and personalized treatment by a RD. Instead, these patients require a universal, non-specialized level of nutrition care to maintain nutritional status. This might look like standardized instructions to meet energy, macro- and micro-nutrient requirements delivered through a handout and/or group class. Patients identified with moderate or suspected malnutrition (SGA B or PG-SGA 4-8), require a short-personalized session with a RD or trained perioperative clinician to provide targeted care based on the specific nutrition-related symptoms (e.g., nausea) that are impeding oral intake. These targeted interventions often require nutrition tips and medical management to sufficiently relieve symptoms to encourage adequate intake. A patient with severe malnutrition (SGA C or PG-SGA ≥ 9) would receive a primary, specialized, one-on-one counseling session and nutrition intervention by a RD. The patient's unique nutrition diagnoses dictate the nutrition intervention. At this stage, nutrition support, including oral supplementation, enteral tube feeding, and parenteral nutrition, is almost always required to optimize nutritional intake in the short window of opportunity before surgery.

In addition to preventing malnutrition and correcting nutrition-identified problems, the nutrition component of a multimodal prehabilitation program should work in synergy with the exercise intervention to support optimal gains in mass, strength, physical fitness, and recovery (10, 40, 82). While resistance exercise is regarded as the main anabolic stimulus, nutrition, including adequate dietary protein, provides the necessary substrate to achieve anabolic gains (83). For a review of nutrition within surgery, we refer the reader to Gillis and Carli (84), for nutrition prehabilitation see Gillis and Wischmeyer (40), and for treating low muscle mass see Prado et al. (85).

### Monitor/Evaluate

Relevant outcome/indicators need to be measured to evaluate whether the nutrition prescription is appropriate and to determine whether progress has been made toward resolving the nutrition diagnosis (1). Estimated protein requirements, for instance, range from 0.8-3 g/kg (Table 2). This is a wide range that requires monitoring to determine whether the prescribed dose is adequate. This step also provides an opportunity to identify barriers [e.g., COM-B questionnaire (86)] and facilitators to support progress, and review/develop new nutrition goals and interventions with patients.

Selection of appropriate outcome/indicators is based on the nutrition diagnosis. As an example, a diagnosis of “inadequate oral intake related to nausea as evidenced by meeting only 50% of estimated energy requirements and 5% weight loss in 1 month” can be monitored with food records and regular weight measurements (see section on Nutrition Assessment) to determine if the diagnosis of inadequate oral intake has worsened, improved, or resolved. Intake-related indicators include nutrient adequacy (e.g., percent energy and protein requirements met), changes in dietary patterns [e.g., healthy eating index (87)], and compliance to prescribed supplements. Biomarkers and biochemical indices can be used to complement intake data. For instance, fructosamine can be used to monitor glycemic control and urinary nitrogen can be used to corroborate protein intake from food records (88). Clinical-related indicators include changes in weight, waist circumference, body composition, and physical function. Patient-related factors include changes in quality of life and knowledge/attitudes related to food and nutrition.

At the targeted level, telephone calls to troubleshoot barriers and asking patients to self-monitor weight can be appropriate. At the specialist level, however, patients require close monitoring and re-assessments so that the nutrition prescription can be modified if it is not adequately meeting patient needs or reaching expected outcomes. While an ideal timeframe for follow-up is unknown, prehabilitation research tends to follow patients weekly or bi-weekly given the short window of opportunity before surgery.

## Applying the Nutrition Care Process Model to Surgical Prehabilitation

Herein, we present three case studies that apply the NCPM to the pre-surgical oncology patient.

### Universal Level of Care

A 65-year-old female presented to her surgeon's office with gynecological cancer. NRS-2002 indicated no recent changes in dietary intake nor changes in weight status (NRS score: 2). Patient was not flagged as having malnutrition risk and thus did not require a RD assessment. To mitigate any future perioperative malnutrition, patient was invited to attend a regularly scheduled weekly pre-operative class that focused on optimizing nutritional intake throughout the perioperative period (before surgery, while in hospital stay, and recovering well at home). The patient was provided information on self-screening and monitoring for malnutrition risk, balanced meals, sample meal plans, and tips to manage common perioperative nutrition-impact symptoms.

### Targeted Level of Care

Assessment: Referral received from preadmission clinic for 59-year-old male diagnosed with colon cancer and duodenal invasion at malnutrition risk (NRS 2002:3). Patient experienced an unintended weight loss of ~3% of his usual stated body weight over the previous month with no weight stabilization. Body mass index classification of overweight status (29.2 kg/m^2^). Total estimated energy and protein intake in 24 h was 74 and 63% of estimated needs, respectively. Patient described inadequate oral intake over the preceding month because of several nutrition-impact symptoms including abdominal pain, diarrhea, reduced appetite, and early satiety. Patient described feeling fatigued, especially upon exertion. Baseline functional assessment indicated that he was physically fit: +0.7 handgrip strength z-score [age and sex-specific z-score (89)], 92% of predicted 6MWT (90) based on age and sex, and 22.5 kg/m^2^ fat-free mass index [<17.0 kg/m^2^ for males indicates reduced fat-free mass (42)]. RD identified that patient is moderately malnourished (SGA: B).

Diagnosis: Inadequate oral intake related to abdominal pain, diarrhea, poor appetite, and early satiety as evidenced by meeting 74% of estimated energy needs, meeting 63% of estimated protein needs, and an unintended 3% weight loss over preceding month.

Intervention: Patient to meet 25 kcal/kg and a minimum of 1.0–1.2 g protein/kg through food intake [ESPEN guidelines (66)]. RD met with patient to assess nutrition knowledge and willingness to change behavior. Patient was provided with targeted dietary tips and handouts to address stated nutrition-impact symptoms and encouragement to support adequate oral intake. Patient-agreed goals: stabilize weight as well as maintain physical fitness and fat-free mass.

Monitor/Evaluation: Follow-up by telephone within 7–10 days to evaluate status of nutrition impact symptoms and oral intake. If nutrition-impact symptoms continue to impede adequate food intake, will assess for oral nutrition supplements (ONS) and medical management of symptoms. Patient to self-monitor weight weekly, if weight does not stabilize, will schedule for one-on-one counseling with a RD.

### Specialist Level of Care

Assessment: Referral received from hepato-pancreato-biliary consultant clinic for 78-year-old female with pancreatic ductal adenocarcinoma at malnutrition risk (MUST score: 4). Patient experienced 19.7% unintended weight loss over 2 months. Body mass index classification of normal weight status (23.6 kg/m^2^). Total estimated energy and protein intake in 24 h was 66 and 43%, respectively. Patient described inadequate oral intake over preceding month because of several nutrition-impact symptoms, including loss of appetite, nausea, taste changes, aversion to food smells, and early satiety. Patient described pale, greasy, oily stool with occasional bloating. Biochemical data indicated low serum vitamin D (18.9 nmol/L; reference value: >50 nmol/L), zinc (6 umol/L; reference value: 10-22 umol/L) and selenium (0.2umol/L; reference value: 0.8-1.5 umol/L). Nutrition-focused physical exam suggested temporalis muscle wasting. Patient described physical limitations, including spending most of day in bed/chair over the past month. Baseline functional assessment was indicative of deficits: −2.0 handgrip strength z-score [age and sex-specific z-score score (89)] and <10 sit-to stands in 30 s [below population norms for age and sex (91)]. RD identified that patient is severely malnourished (SGA:C).

Diagnosis: (1) Severe acute malnutrition related to no appetite, nausea, taste changes, aversion to food smells, early satiety and malabsorption as evidenced by SGA C category, severe weight loss, inadequate protein energy intake, temporalis muscle wasting, and low physical function; (2) Altered gastrointestinal (GI) function related to inadequate pancreatic enzyme replacement therapy as evidenced by steatorrhea and occasional abdominal bloating.

Intervention: Patient to meet minimum of 25 kcal/kg and 1.2 g protein/kg through food intake and oral nutrition supplements [ESPEN guidelines (66)]. RD assessed nutrition knowledge and willingness to change behavior. RD addressed nutrition impact symptoms and encouraged high protein high energy diet through one-on-one counseling. A motility agent was prescribed and instructed to be taken 30 min before meals. Patient was encouraged to consume ONS twice daily (providing an additional 40 g protein and 800 kcal to meet estimated deficit). RD prescribed multivitamin/mineral and vitamin D replacement. Pancreatic enzyme replacement therapy initiated, and education/handouts provided. Patient agreed goals: stabilize/gain weight, improve physical function, improve GI function and nutrient absorption.

Monitor/Evaluate: Patient to record food intake for 3 days (1 weekend day and 2 weekdays) and will reassess total caloric, protein, and ONS intake in 1 week by telephone. Patient to self-monitor weight weekly. Pancreatic enzyme replacement therapy questionnaire and GI symptom rating scale will also be evaluated over telephone in 1 week. Follow up visit scheduled before surgery to re-assess weight, physical function, and readiness /appropriateness to proceed with surgery.

## Conclusion

We have demonstrated, using the nutrition care process, how early coordinated action from surgical and dietary departments can provide optimal nutrition care to pre-surgical patients. Importantly, the NCPM provides a framework to guide professional nutrition practice. Given the recent scoping review of nutrition within prehabilitation research (7), which indicated that many nutrition interventions are currently conducted without reference to best practice guidelines, we suggest that implementation of the systematic NCPM could enhance the contribution of nutrition to prehabilitation and improve patient outcomes.

## Author Contributions

All authors have made substantial contributions to the manuscript including editing and approval of the final manuscript.

## Conflict of Interest

The authors declare that the research was conducted in the absence of any commercial or financial relationships that could be construed as a potential conflict of interest.
